# Case Reports of Teprotumumab as Treatment for Monoclonal Antibody-Induced Thyroid Orbitopathy

**DOI:** 10.3390/reports8040246

**Published:** 2025-11-26

**Authors:** Jared Moon, Nicole Duncan, Jeff Yorio, Ethan Meltzer, Moe H. Aung

**Affiliations:** 1 Mitchel and Shannon Wong Eye Institute, Department of Ophthalmology, Dell Medical School at the University of Texas at Austin, Austin, TX 78712, USA; 2 Texas Oncology, Austin, TX 78731, USA; 3 Department of Neurology, Dell Medical School at the University of Texas at Austin, Austin, TX 78712, USA

**Keywords:** nivolumab, alemtuzumab, teprotumumab, thyroid eye disease, immune related adverse events, orbitopathy

## Abstract

**Background and Clinical Significance**: Targeted biologic therapies, especially monoclonal antibodies (mAbs) such as nivolumab and alemtuzumab, have revolutionized treatment for malignancies and autoimmune conditions but can cause rare immune-related adverse events (IRAEs), including orbitopathy. To date, only a handful of cases have described the treatment of thyroid eye disease secondary to mAbs, and even fewer have described how to treat refractory disease. **Case Presentation**: We are illustrating two cases in this report: a 73-year-old woman who developed thyroid eye disease (TED) after nivolumab therapy for melanoma, and a 36-year-old man who presented with TED following alemtuzumab treatment for multiple sclerosis. Both patients failed corticosteroid therapy but showed a significant improvement with teprotumumab, an anti-insulin-like growth factor (IGF)-1 receptor mAb. **Conclusions**: These cases highlight underrecognized orbital IRAEs linked to mAb therapy and demonstrate teprotumumab’s potential as an effective option for steroid-refractory thyroid orbitopathy. Clinicians should maintain an awareness of orbital complications in patients receiving mAbs to enable prompt diagnosis and intervention, minimizing visual morbidity. Further studies are needed to clarify the pathogenesis of mAb-associated orbitopathy and to establish evidence-based treatment protocols for these rare but impactful complications.

## 1. Introduction and Clinical Significance

Targeted biologic therapies have quickly become a mainstay in modern medicine. By inhibiting specific immune pathways, they offer the potential for greater precision, fewer unintended off-target adverse effects, and improved outcomes compared to traditional systemic treatments. In particular, the development of monoclonal antibodies (mAbs) for medical use has grown dramatically in the past three decades, with over 80 FDA-approved agents since the first one in 1986 [1]. Amongst these, immune checkpoint inhibitors (ICIs), such as nivolumab, have revolutionized oncology therapy, particularly in metastatic melanoma. Similarly, targeted biologic therapies against B and/or T lymphocytes, such as alemtuzumab, in multiple sclerosis have demonstrated a dramatic effect in reducing relapse rates and long-term disability outcomes. However, the use of these mAbs has been associated with immune-related adverse events (IRAEs), which can affect virtually any organ system, including the eyes. Fortunately, orbital complications remain rare but have been underrecognized.

Clinical Significance: The emergence of orbital inflammatory complications associated with targeted biologic therapies—especially immune checkpoint inhibitors (ICIs)—highlights an underrecognized yet potentially vision-threatening adverse event. Though ocular and orbital immune-related adverse events (IRAEs) are rare, their manifestations (e.g., orbital myositis, thyroid eye disease (TED), uveitis) can occur months to years after therapy cessation and may lead to irreversible damage if not promptly diagnosed and treated. Multidisciplinary collaboration and baseline ocular assessment are essential.

## 2. Case Presentation

In this report, we present two patients who developed orbitopathy related to mAb therapy. The first is a 73-year-old woman who presented with progressive binocular diplopia and was found to have TED after nivolumab treatment for melanoma. The second is a 36-year-old man with relapsing–remitting multiple sclerosis who developed TED following alemtuzumab therapy. Both responded well to teprotumumab after failing corticosteroid treatment. All procedures were conducted in compliance with the Health Insurance Portability and Accountability Act (HIPAA) guidelines and adhered to the principles of the Declaration of Helsinki. Both cases were published anonymously with the patient’s informed consent.

### 2.1. Case 1

A 73-year-old woman presented with progressive binocular oblique diplopia. She had a remote history of a right orbital floor fracture, which was surgically repaired (years before current presentation) with no report of symptomatic diplopia nor observed restrictive eye movement at that time. She was also diagnosed with stage IIIB melanoma and treated previously with a wide local excision and 11 cycles of nivolumab. However, nivolumab therapy was discontinued when the patient developed adrenal insufficiency, thought to be triggered by immune checkpoint inhibition. During the treatment with nivolumab, she also developed immune-mediated thyroiditis with a peak thyroid-stimulating hormone (TSH) of 13.56 (uIU/mL) but low free T4, at 0.66 (NG/dL), prompting the initiation of levothyroxine.

**Figure 1:**
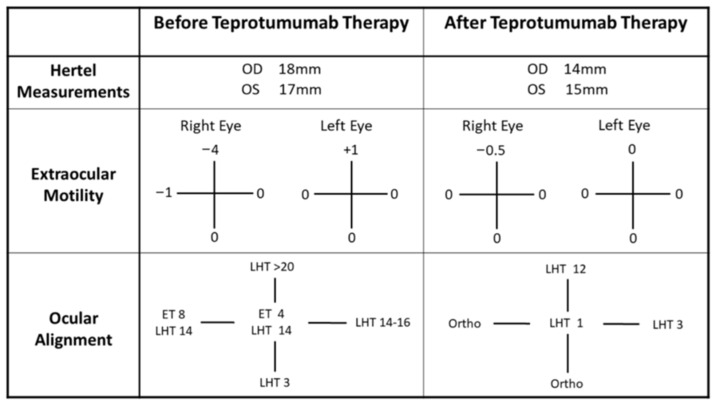
Comparison of pertinent neuro-ophthalmic exam findings before and after teprotumumab treatment for our first case. Before the teprotumumab therapy (second column), patient had relative proptosis in both eyes, worse in the right eye than the left eye. She also exhibited significantly limited supraduction and mild abduction deficit of her right eye. This resulted in incomitant ocular misalignment with esotropia and left hypertropia (or right hypotropia). Her esotropia was worse at right gaze and her left hypertropia (or right hypotropia) was worse at up gaze, corresponding to her eye motility dysfunctions. After completion of teprotumumab therapy (third column), patient showed reduced proptosis. The restriction in her extraocular movements almost completely resolved with mild residual supraduction deficit in her right eye. She still has incomitant left hypertropia (or right hypotropia) but with less severity. Subjectively, she was able to compensate for the trace left hyperphoria at primary gaze, and denied binocular diplopia.

**Figure 2:**
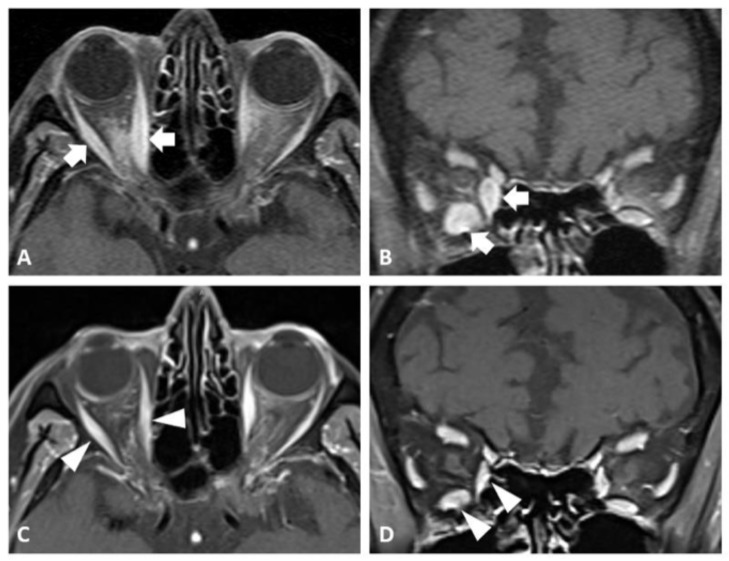
(**A**,**B**) MRI T1 sequence with contrast images of patient’s orbits before teprotumumab treatment. In both (**A**) axial and (**B**) coronal sections, there is asymmetric enlargement of the right inferior and medial rectus musculature, and to a lesser extent involvement of the lateral rectus muscle, as indicated by the white arrows. (**C**,**D**) MRI T1 sequence with contrast images of patient’s orbits after teprotumumab treatment taken about 1 year from initial MRI and about 3 months after completion of teprotumumab therapy. In both (**C**) axial and (**D**) coronal images, there is interval improvement in the sizes of the right inferior, medial, and lateral muscles, as indicated by the white arrowheads.

On presentation to neuro-ophthalmology (about four years post-nivolumab therapy), her visual acuity was 20/20 bilaterally with intact color vision and no afferent pupillary defect. The posterior segment was within normal limits, with healthy-appearing optic nerves. However, she demonstrated a restrictive motility in the supraduction and abduction of her right eye. She also had proptosis, left upper eyelid retraction (MRD1 6 mm left eye), and incomitant ocular misalignment (Figure 1). Her clinical activity score (CAS) on presentation was 3/7 based on the inflammation of the plica, restricted extraocular movements, and eyelid swelling, indicating disease activity. Magnetic resonance imaging (MRI) of the orbits showed an asymmetric enlargement of the right medial and inferior rectus muscles consistent with thyroid orbitopathy (Figure 2A,B). A brain MRI was unremarkable for intracranial pathology that can cause ocular misalignment. Subsequent testing revealed low TSH (0.058 uIU/mL) but normal T3 and T4 (3.2 and 1.69 NG/dL, respectively) levels, along with elevated thyroid-stimulating immunoglobulin (TSI) (1.61 IU/L). These findings were consistent with TED in a euthyroid patient, likely related to prior nivolumab therapy.

We initiated a 12-week course of intravenous (IV) methylprednisolone (500 mg once weekly for 6 weeks, followed by 250 mg once weekly for another 6 weeks)—based on the improvement shown in prior reported orbitopathy cases due to nivolumab after either IV or oral steroid treatment [2]. Despite this, there was no clinical improvement for our patient. Given the persistent diplopia and progressive orbitopathy, the decision was made to start teprotumumab (TEPEZZA^®^, Amgen Inc., Thousand Oaks, CA, USA). After three infusions, the patient reported symptomatic improvement. By the end of treatment (completion of eight sessions), her subjective diplopia had resolved. A subsequent examination showed a reduction in her proptosis as well as an improvement in ocular motility and alignment (Figure 1). A follow-up MRI of her orbits confirmed a reduction in extraocular muscle enlargement and proptosis (Figure 2C,D).

### 2.2. Case 2

A 36-year-old man with a history of relapsing–remitting multiple sclerosis presented with symptomatic proptosis and diplopia. He had been treated with two courses of alemtuzumab (LEMTRADA^®^, Genzyme, Cambridge, MA, USA) three years earlier. He subsequently developed subclinical hyperthyroidism (TSH 0.04 uIU/mL, free T4 1.7 NG/dL), later diagnosed as Graves’ disease, a manifestation of alemtuzumab-induced autoimmune thyroid dysfunction. Despite management with methimazole, he still developed ocular symptoms.

**Figure 3:**
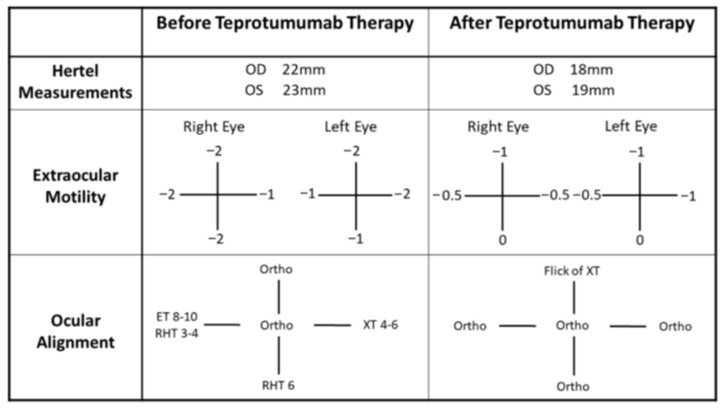
Comparison of pertinent neuro-ophthalmic exam findings before and after teprotumumab treatment for our second case. Before the teprotumumab therapy (second column), patient had exophthalmos in both eyes. He also exhibited significant restrictions in eye motility in both eyes. This resulted in incomitant ocular misalignment. After completion of teprotumumab therapy (third column), patient showed reduced proptosis in both eyes. The restriction in his extraocular movements also improved greatly with only some residual supraduction and lateral restricted eye movement in both eyes. He was orthophoric at almost all gazes except trace exophoria at up gaze. Fortunately, he was largely orthophoric at primary gaze throughout.

**Figure 4:**
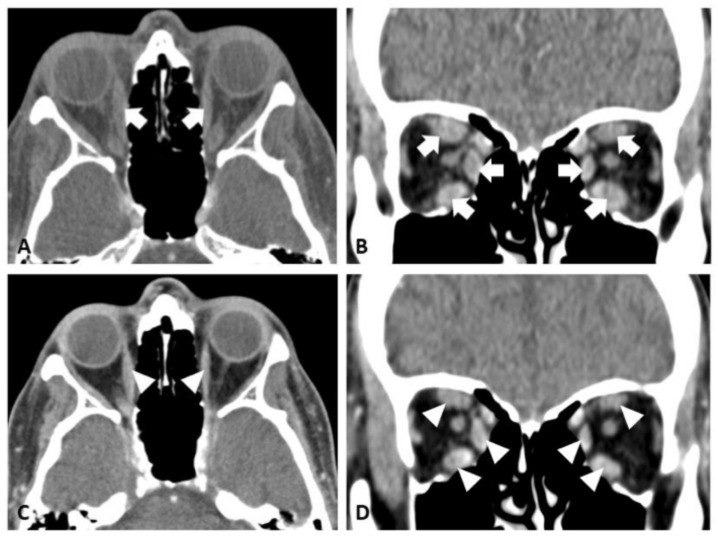
(**A**,**B**) CT images of patient’s orbits before teprotumumab treatment. In both (**A**) axial and (**B**) coronal sections, there is enlargement of the extraocular muscles (sparing the lateral rectus muscles) in both eyes, as indicated by the white arrows. (**C**,**D**) CT images of patient’s orbits after teprotumumab treatment taken about 1.5 year from the initial CT study and about 3 months after completion of teprotumumab therapy. In both (**C**) axial and (**D**) coronal images, there is interval-reduced enlargement of the extraocular muscles bilaterally, as indicated by the white arrowheads.

On presentation to neuro-ophthalmology (about three years post-alemtuzumab therapy), his visual acuity was 20/20 in both eyes, with normal color vision and no afferent pupillary defect. Like our first case, a fundus exam showed healthy optic nerves with no evidence of compressive optic neuropathy. However, the remaining exam revealed bilateral proptosis, mechanical restriction, and incomitant strabismus (Figure 3). His CAS was 3/7 based on eyelid swelling, conjunctival injection, and plica inflammation, indicating disease activity. Computed tomography (CT) of the orbits demonstrated an enlargement of all rectus muscles except the lateral rectus, along with increased retrobulbar fat (Figure 4A,B). TSI (6.95 IU/L), thyrotropin receptor antibody (TRAb) (21.11 IU/L), and thyroglobulin antibody (730 IU/mL) levels were all elevated. Serologic testing for myasthenia gravis as a possible alternative etiology was negative as well. Given these results, he was diagnosed with TED, likely related to prior alemtuzumab therapy.

Despite a 12-week course of IV methylprednisolone therapy (500 mg once weekly for 6 weeks, followed by 250 mg once weekly for another 6 weeks), his symptoms did not improve. Teprotumumab therapy was therefore initiated, and the patient completed eight infusion sessions. Following teprotumumab treatment, the patient reported the resolution of diplopia and significant reduction in proptosis. A repeat examination showed a significant reduction in his exophthalmos, nearly normal extraocular movement with only mild restriction, and a much improved ocular alignment (Figure 3). Repeat CT orbits corroborated the reduced extraocular muscle inflammation and enlargement (Figure 4C,D).

## 3. Discussion

In this report, we presented two cases of symptomatic TED triggered after prior treatment with mAb: the first case after nivolumab and second case after alemtuzumab. Thyroid eye disease developing 3–4 years after nivolumab and alemtuzumab is still very plausibly immune-related, since both drugs did cause thyroid dysfunction for our two patients during the treatment phase. Even though the orbitopathy came later, the peri-treatment thyroid autoimmunity shows a plausible mechanistic link. Moreover, a prior systematic review and meta analysis showed that the mean time to thyroid-associated orbitopathy due to alemtuzumab could be as late as 37 months after treatment completion [3]. The delayed onset suggests that the mechanism is likely due to immune reconstitution dynamics. Though conventional corticosteroid therapy was not effective for both of our patients, a subsequent treatment with teprotumumab resulted in a dramatic improvement of their TED, as evidenced by the reduced exophthalmos, extraocular dysmotility, and ocular misalignment.

Nivolumab is an mAb targeting the programmed cell death protein (PD-1) that has transformed the treatment of various malignancies, including lung cancer and melanoma. It is amongst a class of biologics that inhibit immune checkpoints, such as PD-1 and cytotoxic T-lymphocyte-associated protein-4 (CTLA-4). However, as these checkpoints play essential roles in maintaining peripheral tolerance, blocking them with ICIs can inadvertently trigger autoimmune responses affecting multiple organ systems, including the eyes [4,5,6,7]. Ocular IRAEs occur in approximately 1% of patients on ICIs and can present as dry eye, uveitis, scleritis, optic neuropathy, or orbital inflammation [7,8]. The greater immune activation associated with the CTLA-4 blockade may explain the higher incidence of reported ocular IRAEs compared to PD-1 medications [9,10,11]. CTLA-4 gene polymorphisms have been linked to both Graves’ disease and TED, supporting a potential pathogenic role [12,13,14,15,16]. Our first patient case is one of only a handful of reported cases of TED induced by nivolumab monotherapy [4,5,6]. Future studies should explore genetic predispositions to mAb-associated orbitopathy.

Alemtuzumab is an mAb targeting CD52, which has been used for relapsing–remitting multiple sclerosis, certain rheumatologic disorders, and hematologic malignancies [17,18]. CD52 is a cell surface protein expressed by mature lymphocytes that can be targeted for mAb therapy. The inhibition of CD52 with alemtuzumab depletes circulating lymphocytes, which inadvertently carries a high risk of secondary autoimmunity. Specifically, up to 33–36% of treated patients can develop autoimmune thyroid disease (AITD) [19,20,21,22]. The underlying mechanism of alemtuzumab inducing AITD, which occurs after immune reconstitution, remains unknown. As B cell repopulation outpaces T cell recovery, a pro-autoimmune environment emerges—further driven by elevated IL-21 cytokines. This can lead to autoreactive lymphocytes and the production of TSI [23,24]. TED may develop in a subset of these cases, with recent estimates suggesting an incidence of 13–16% [25,26]. Interestingly, AITD is more common in patients with multiple sclerosis than in those treated for other conditions, such as rheumatoid arthritis, suggesting a disease-specific predisposition [27,28,29]. To date, 44 cases of autoimmune thyroid eye disease (AITED) have been reported. Management generally mirrors that of conventional TED. Mild disease is treated with topical lubrication and lifestyle modifications (e.g., smoking cessation), while moderate to severe disease may require radioiodine therapy, surgery, corticosteroids, or targeted biologics. Several reports have described the successful off-label use of rituximab, tocilizumab, and teprotumumab in this setting, including our case [22,26,30,31,32].

Teprotumumab, an anti-IGF-1R mAb approved for TED, has shown a strong efficacy in reducing proptosis and inflammation while improving quality of life [33]. Although not currently approved specifically for ICI-induced or alemtuzumab-triggered TED, shared pathophysiologic mechanisms support its potential utility in this setting. Our cases add to a growing body of evidence suggesting that teprotumumab can be effective for steroid-refractory orbitopathy linked to mAb therapies. Larger studies are warranted to determine its role in these rare presentations.

## 4. Conclusions

Our cases here provide additional evidence of TED manifestation due to mAb usage and present teprotumumab as a viable treatment option for refractory disease. Clinicians should remain vigilant for orbitopathy in patients receiving mAb therapies, particularly when ocular symptoms arise. Early recognition and treatment may prevent permanent visual sequelae. More research is needed to characterize these rare complications and to guide evidence-based management strategies.

## Data Availability

The original contributions presented in this study are included in the article. Further inquiries can be directed to the corresponding author.

## Abbreviations

The following abbreviations are used in this manuscript:

TED	Thyroid Eye Disease
IRAE	Immune-Related Adverse Events
mAb	Monoclonal Antibodies
IGF	Insulin-Like Growth Factor
TSH	Thyroid-Stimulating Hormone
CAS	Clinical Activity Score
MRI	Magnetic Resonance Imaging
TSI	Thyroid-Stimulating Immunoglobulin
IV	Intravenous
CT	Computed Tomography
TRAb	Thyrotropin Receptor Antibody
PD-1	Programmed Cell Death Protein 1
CTLA-4	Cytotoxic Lymphocyte-Associated Protein-4
AITD	Autoimmune Thyroid Disease
AITED	Autoimmune Thyroid Eye Disease
